# Modern Learning from Big Data in Critical Care: Primum Non Nocere

**DOI:** 10.1007/s12028-022-01510-6

**Published:** 2022-05-05

**Authors:** Benjamin Y. Gravesteijn, Ewout W. Steyerberg, Hester F. Lingsma

**Affiliations:** 1 Department of Public Health, Erasmus University Medical Center, Doctor Molewaterplein 40, 3015 GD Rotterdam, Netherlands; 2 Department of Biomedical Data Sciences, Leiden University Medical Center, Leiden, Netherlands

**Keywords:** Observational data, Machine learning, Statistics, Prediction, Clustering

## Abstract

Large and complex data sets are increasingly available for research in critical care. To analyze these data, researchers use techniques commonly referred to as statistical learning or machine learning (ML). The latter is known for large successes in the field of diagnostics, for example, by identification of radiological anomalies. In other research areas, such as clustering and prediction studies, there is more discussion regarding the benefit and efficiency of ML techniques compared with statistical learning. In this viewpoint, we aim to explain commonly used statistical learning and ML techniques and provide guidance for responsible use in the case of clustering and prediction questions in critical care. Clustering studies have been increasingly popular in critical care research, aiming to inform how patients can be characterized, classified, or treated differently. An important challenge for clustering studies is to ensure and assess generalizability. This limits the application of findings in these studies toward individual patients. In the case of predictive questions, there is much discussion as to what algorithm should be used to most accurately predict outcome. Aspects that determine usefulness of ML, compared with statistical techniques, include the volume of the data, the dimensionality of the preferred model, and the extent of missing data. There are areas in which modern ML methods may be preferred. However, efforts should be made to implement statistical frameworks (e.g., for dealing with missing data or measurement error, both omnipresent in clinical data) in ML methods. To conclude, there are important opportunities but also pitfalls to consider when performing clustering or predictive studies with ML techniques. We advocate careful valuation of new data-driven findings. More interaction is needed between the engineer mindset of experts in ML methods, the insight in bias of epidemiologists, and the probabilistic thinking of statisticians to extract as much information and knowledge from data as possible, while avoiding harm.

**Supplementary Information:**

The online version contains supplementary material available at 10.1007/s12028-022-01510-6.

## Introduction

Because of technical capacity and scientific interest, data sets are becoming increasingly large in medical research. In critical care, this is even more apparent: there is an abundance of continuously measured clinical, laboratory, radiological, and pharmaceutical data in the intensive care unit. In neurocritical care specifically, we have seen a vast increase of parameters that can be measured. Whereas we traditionally were only able to record systemic metrics, such as blood pressure and heart rate, nowadays we are able to (invasively) monitor highly granular physiological parameters, such as local brain oxygen pressure or intracerebral glucose and lactate levels [1, 2]. Already, multiple initiatives exist that share such anonymized intensive care unit data freely for research purposes [3–8]. Also, large observational studies and registries have been rolled out during the last decades, for example, in traumatic brain injury (TBI), [9–12], that make their data available for researchers on request. This omnipresence of data provides an opportunity to improve on clinical care [13]. However, it remains a challenge to inform treatment decisions based on the volume and dimensionality of big data to improve patient outcomes [2].

Within this context, it is important to realize that the scientific method seems to gradually shift from research question as a starting point towards available data as a starting point [14]. In the latter approach, there is less selection in relationships being tested and the probability is lower  that the findings are actually true [15]. Three types of research questions can be answered by analyzing data, increasing complexity: descriptive, predictive, and causal questions [16].

To answer such research questions, researchers can apply either more traditional statistical techniques or more modern machine learning (ML) algorithms. Part of the enthusiasm for ML algorithms stems from the promising results in diagnostic research. For neurocritical care, examples include deep learning algorithms to identify brain lesions [17–19] and perform volumetrical analyses [20]. The performance of these algorithms for these types of descriptive questions seems reliable and high and therefore likely of great assistance to assist or automatize interpretation of visual diagnostic information.

However, there is more discussion as to how ML algorithms should be applied and interpreted for clustering (a type of descriptive question) and for predictive questions. Inadequate predictive algorithms can cause harm when implemented. This was the case for the Epic Sepsis Model; because this model was inadequately validated before implementation, the model caused alarm fatigue and underdiagnosis of sepsis in clinical practice [21].

In this article, we aim to explain characteristics of commonly used data analysis techniques and to present a perspective on responsible use of these techniques for answering clustering (descriptive) and prediction questions. Effectively, this article provides guidance for clinically oriented readers to avoid the necessity to follow methodological literature [22]. It is important for clinicians to judge the appropriateness of published algorithms because invalid algorithms that are used to inform clinical practice might ultimately harm patients.

## Data Analysis Techniques

Researchers can use various techniques to answer descriptive, predictive, and causal questions in their data sets [16]. A selection of most commonly used techniques is provided in Table 1, and these have been described elsewhere in more detail [23–40]. Although there is a tendency to classify these techniques as ML versus statistical techniques, there are many common characteristics. We therefore refrain from referring to these algorithms in a dichotomous way because the distinction is not very clear. First and foremost, they are all algorithms that, when provided with appropriate data, quantify relationships between variables. The most fundamental differences between the techniques are in flexibility and functional aim. For more detailed descriptions of terms found in this article, we refer to Online Appendix.

**Table 1:** Frequently used algorithms for modeling big data

Algorithm	Commonly referred to as^a^	Degree of flexibility	Functional aim^b^
Classic regression [23, 24, 33]	Statistical learning	Relatively limited but can be extended with nonlinear terms, interactions, mixed effects	Y|X
Bayesian regression [34, 35]	Statistical learning	Moderately, can also be extended with nonlinear terms, interactions, mixed effects	Y|X
Penalized regression [36, 37]	Statistical learning	Moderately flexible, can also be extended with nonlinear, interactions, mixed effects	Y|X
Neural network [39, 40]	Machine learning (supervised)	Very flexible, with various structural architectures and functionalities	Y|X
Classification and regression tree [25]	Machine learning (supervised)	Limited flexibility	Y|X
Random forest [26]	Machine learning (supervised)	Moderately flexible	Y|X
Gradient boosting machine [27]	Machine learning (supervised)	Moderately flexible	Y|X
Support vector machine [28]	Machine learning (supervised)	Moderately flexible, with many available kernels possible	Y|X
Super learner [29, 30]	Machine learning (supervised)	Very flexible: cumulative flexibility of all underlying models	Y|X
Clustering [31]	Machine learning (unsupervised)	Relatively limited but can be extended for various types of data (continuous, categorical, or mixed)	X
Latent class analysis [32]	Statistical learning	Relatively limited but can be extended for various types of data (continuous, categorical, or mixed)	X|Y

^a^No clear definitions available, our opinion

^b^| = notation for conditionality; thus, Y|X means “Y given X”

**Fig. 1:**
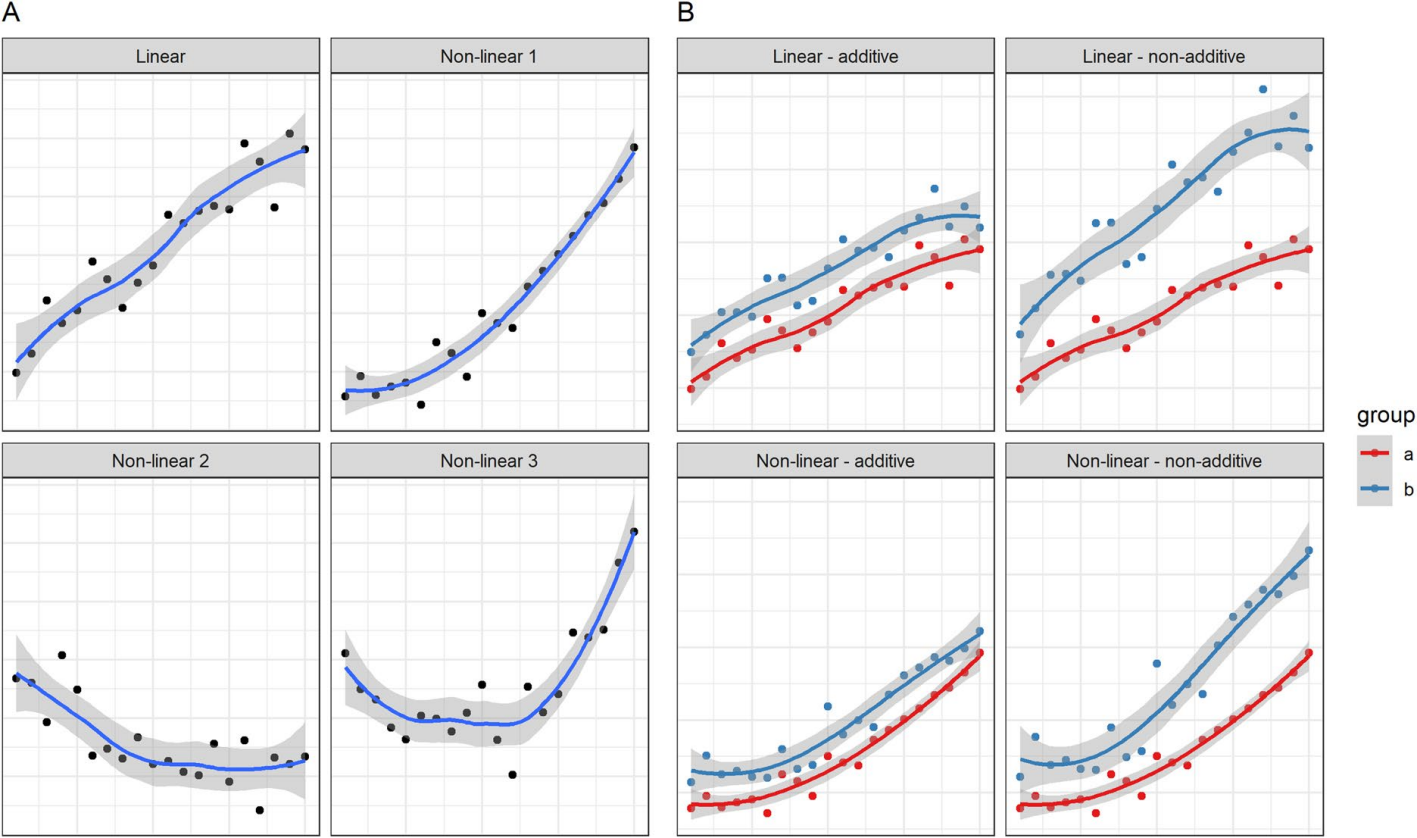
Illustration of different types of relationships. **a,** Various ways of how two variables can be related linearly (upper left subpanel) or nonlinearly (the other subpanels). The data on the *x*-axis is an arbitrarily chosen range of numbers, and the relationship with the *y*-data was artificially simulated, including some noise (random error). **b,** the concept of nonadditivity. The upper two subpanels show for a linear relationship how the effect of group (color) can be additive (left) or nonadditive (right) over the *x*-variable. The bottom subpanels show the same for a nonlinear relationship

Flexibility is the ability of a model to incorporate various types of relationships. Relationships can be linear or nonlinear and additive or nonadditive (Fig. 1) [41].

**Fig. 2:**
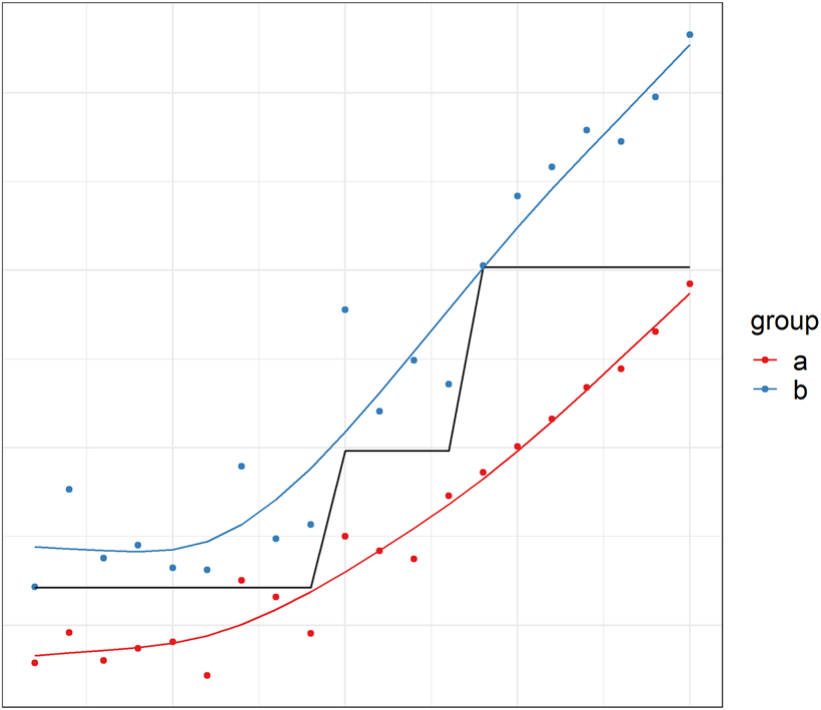
Fitting a regression model (lm function in R) and a regression tree (rpart function in R) to the nonadditive, nonlinear relationship shown in Fig. 1b, bottom right subpanel. Again, the data shown on the *x*-axis was an arbitrarily chosen range of numbers, and the *y*-data were artificially simulated, including some noise (random error). The regression model (in colored lines) included a restricted cubic spline and an interaction term between group and x and follows the relationship nicely. The regression tree (black line) failed to include group in the final tree and only included x, thereby disregarding complexity in the data

Nonlinear associations are, for example, the U-shaped effect of blood pressure on mortality in trauma patients with active hemorrhages [42] or the effect of body mass index on mortality in the general population [43]. Nonlinear associations are abundant in nature. Most algorithms can be extended to include these relationships. For example, in classical regression, nonlinear relationships can be explored by adding polynomial terms, such as x^2^. Further exploration may be with the log transformation, as is commonly used for laboratory measurements [44], or splines [23]. A neural network with hidden layers includes nonlinearity implicitly through its complex architecture. On the contrary, classification and regression trees do not really include nonlinear relationships (Fig. 2).

More rarer is the notion of nonadditivity [45, 46], commonly referred to as interaction (Fig. 1). We call two effects nonadditive if the association of a variable with the outcome differs when another variable changes its value. A recent example was the CRASH-3 trial, which showed that tranexamic acid reduces head-injury-related deaths, primarily in very early administration [47]. The effect of tranexamic acid is dependent on time; therefore, these effects are nonadditive. Again, classical regression can be extended to include interaction effects [23], and neural networks do so implicitly, in contrast to classification and regression tree models (Fig. 2).

The functional aim refers to how the variables in the algorithm are linked together. Researchers are required to define this (to various extent per method) before performing the analysis. Given the three types of possible research questions [16], data can be either baseline characteristics (“X”) or outcome (“Y”). Algorithms vary in the way they couple “X” with “Y” (Table 1), which determine what types of research questions they might feasibly answer, given appropriate data.

## Clustering

Clustering and latent class analysis are techniques that focus on relating “X” characteristics. They are useful to answer descriptive questions. Some clustering algorithms also consider the “Y” outcome, such as latent class models. They can identify clusters conditional on outcome (“X|Y”).

**Fig. 3:**
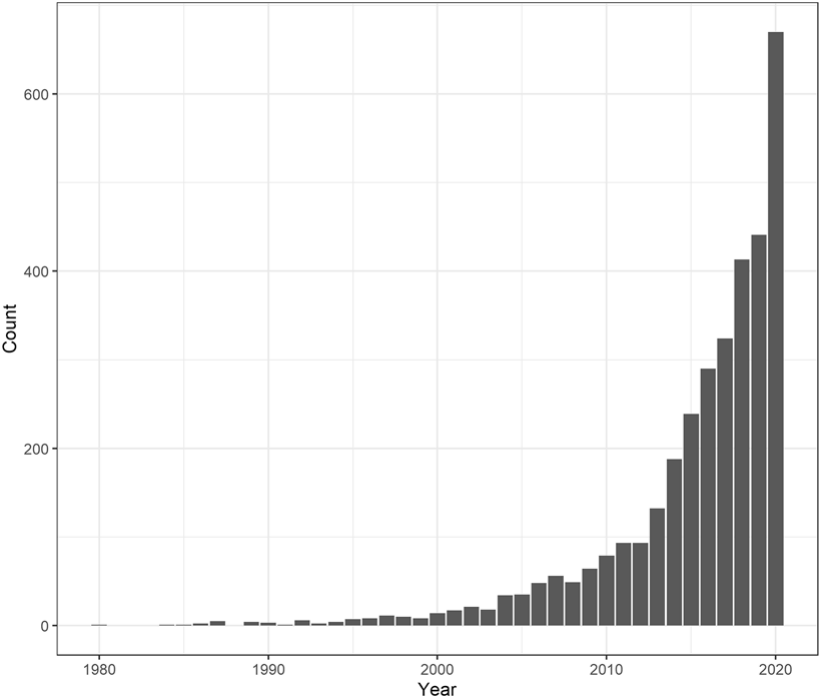
The increase in popularity of clustering studies in critical care. We used as a search string “(clustering OR unsupervised OR hypothesis-free) AND critical care” and included studies in Pubmed up to 2020

In critical care, these techniques have been increasingly popular to identify subgroups or “clinical phenotypes” of patients (Fig. 3). The premise is that by identifying these clinical phenotypes, we learn something intrinsic to that population that informs us how to better describe or classify patients or better allocate treatment. Clustering studies in (neuro)critical care summarize the patterns in high-dimensionality of neurocritical care data [48–50]. Clustering algorithms commonly are performed in a static way with baseline data and/or outcome data. Examples are rare when continuously reported vital parameters (a characteristic of neurocritical care data) are incorporated in the analyses in a more dynamic way.

An important challenge for clustering studies is to ensure and assess validity. There are various useful measures for internal validity that assess how appropriate the clusters are formed within a data set [51–54]. External validity, defined as the degree of how well the identified clusters can be applied to new data sets or new patients, is harder to assess; clustering focuses on accurately describing the current data in relevant groups. Because it does not calculate a general metric or parameter of how the clusters depend on data values, new data or new patients cannot be clustered into the identified groups. It is, however, possible to repeat the clustering analysis in a different data set with the same number of clusters and variables [55] and assess whether the clusters seem similar. More problematic is to apply these clusters to a single patient. For example, given that a patient has a Glasgow Coma Scale (GCS) score of 9, has extracranial injury, and has had a low energy trauma mechanism [56], one can only relate these patient characteristics to the average characteristics of the clusters. We need to make an educated guess which of the clusters most resembles our patient. Therefore, the clinical usefulness of clustering patient groups remains limited, for now.

Interestingly, after having identified clusters, researchers commonly describe these clusters again based on the outcome. For example, clusters of patients with COVID-19 have been described on the mortality as observed by cluster [50, 55], clusters of patients with TBI have been described by the associated functional outcome [56, 57], and clusters of neurocritical care patients with invasive neuromonitoring “may have implications for…outcome predictions” [58]. From a prognostication perspective, this is inefficient; by first categorizing “X,” researchers lose information to predict “Y” [59, 60]. Therefore, predictive questions commonly require different techniques.

## Prediction

Predictive research involves the development and validation of predictive algorithms [61–64]. The primary aim of predictive research is to most accurately predict outcome. All algorithms, ML techniques and statistical techniques, that couple “X” to “Y” are potentially suited for prediction (Table 1).

## Development

**Fig. 4:**
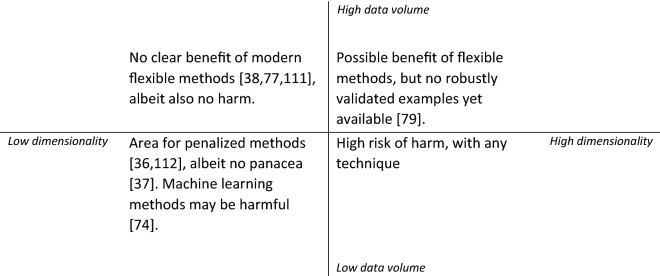
Areas in which different types of algorithms might be considered for predictive modeling

We discern two characteristics of data sets that are relevant for researchers when they want to decide on a technique to develop a predictive algorithm (Fig. 4). On the one hand, dimensionality of the data is relevant, which is the number of potentially relevant predictors in the predictive algorithm. On the other hand, volume of the data is relevant. Volume of data entails the total number of patients and, specifically, the number of events of the least occurring outcome in case of dichotomous outcomes [65]. An example of low dimensionality and low volume is the Ottawa ankle rule [66]. This decision rule informs whether patients with ankle injuries need an x-ray. This rule was developed in a data set with 70 events in 689 patients, and includes five predictors (event per variable [EPV] = 70/5 = 14). An example of high dimensionality and high volume is the OHDSI (Observational Health Data Sciences and Informatics) model to predict hemorrhagic transformation in patients with ischemic stroke [67]. This model includes 612 predictors and was developed in electronic health record data with 5,624 events in 621,178 patients with stroke (EPV = 5634/612 = 9). Note that although the latter model is developed in a much larger cohort with many more events, the number of EPV is lower. The model is therefore not free from risk of overfitting [68]. We discuss the application of statistical techniques and ML techniques in four areas that can be defined by these two characteristics (dimensionality versus volume; Fig. 4).

First, with low-volume and high-dimensional data, it may be unwise to perform any predictive research. The risk of overfitting is too large [62], resulting in potentially harmful prediction tools [69, 70]. For example, a systematic review of ML in routinely collected intensive care unit data estimated that 30% of support vector machines were trained on fewer than 100 patients [71]. The chosen technique does not matter much. Both for regression methods as well as for ML techniques, adequately large sample sizes are necessary [72, 73]. However, because ML techniques are data hungry [74], using these techniques will likely result in more invalid or inaccurate prediction in this setting. Therefore, these techniques may be more harmful than traditional statistical methods in this setting.

Second, with low-volume data and low-dimensional data, we suggest using methods that focus on stability of estimation of the model (in either method). Exemplary prediction models that have been developed in this area are prediction models for acquired weakness in the intensive care unit (8–25 candidate predictors and 25–190 events) [75]. Within this area, penalized regression techniques are often used to shrink the coefficients during the estimation of the model [36, 76]. Therefore, they limit the extent to which extreme coefficients are estimated. This limits the effect of “overfitting”: the problem that the developed model performs well in the original data set but worse when applied to new data sets or to real patients. Neural networks can also use methods to avoid overfitting. Examples of methods to limit overfitting in neural networks are penalizing estimated weights (called “regularization”) or setting weights back to zero at random (called “drop-out”) [40]. It is important to realize that all abovementioned methods work poorer in smaller data sets in the sense that it is difficult to estimate the penalty parameter reliably [37]. Although it is appropriate to use these methods when sample size is limited, it is preferable to have a larger sample size. Unfortunately, as can be seen for the example of prediction models for acquired weakness in the intensive care unit, these techniques are underused [75].

Third, the area in which data volume is large and dimensionality is low is a safer area for predictive research. In contrast with the previous setting, there is less risk of overfitting. ML techniques and statistical techniques seem to perform similarly within this context [38, 77]. For example, only small differences in performance (discrimination, calibration) were found between various algorithms to predict outcome in TBI [77]. A possible explanation is that within the context of clinical data, most predictor effects are largely linear and additive on an appropriate modeling scale [62, 78]. Therefore, more flexible methods have limited opportunity to improve their predictions.

Fourth, the area of high volume and high dimensionality seems the most appropriate area for ML techniques. The previously mentioned OHDSI model to predict hemorrhagic transformation in patients with ischemic stroke is such an example in neurocritical care [67]. The increased flexibility can potentially exploit subtle nonadditive or nonlinear effects to improve accuracy of predictions. Unfortunately, current published predictive studies with ML techniques remain at high risk of bias [38, 79]. Finally, although sample size might seem larger within this context, we should remain aware of the number of EPV, indicating the effective sample size.

To summarize, the two suggested characteristics (dimensionality and volume) can be useful to inform what type of flexibility or control is required of an algorithm to be used reliably in a specific context. With reliability¸ we here mean that there is a high likelihood that the model performs similarly well (in terms of discrimination and calibration) in a different context. To actually confirm whether the algorithm performs well in a different context, researchers do need to rigorously validate predictive algorithms (see following section) [80].

For neurocritical care, another interpretation of dimensionality may be used to inform what algorithm can be used for a predictive question. Dimensionality may also refer to the extent to which continuous measurements (e.g., intracranial pressure, local brain oxygen levels) need to be incorporated into the algorithm. Although repeated measurements are increasingly common in neurocritical care, their inclusion in predictive algorithms can be improved [81]. To adequately model repeated measurements, specific techniques are required, such as mixed effects regression [82], joint modeling [83], or recurrent neural networks [40].

## Validation

To ensure reliable applicability in clinical practice, predictive algorithms require extensive validation [61]. There are various ways of validating a predictive algorithm. In order of rigorousness, researchers can perform internal, internal–external, and external validation [80]. When risk of bias is high, performance is likely overestimated [84].

It is common in ML studies to use “split sample validation” as an internal validation method. We train the model in a training set and then estimate performance in the test set. This method is, however, quite inefficient to estimate performance [85]. Better methods are, for example, cross-validation or bootstrap resampling [23].

A more exiting variant of cross-validation is internal–external validation [77, 80]. This method can be used in data sets with multiple clusters (e.g., multiple centers, multiple studies). The algorithm is developed on all but one cluster and tested in the set that was withheld. This is repeated until all data sets have been used as the validation set and all estimated performances are averaged.

The most robust way to estimate performance is in a data set in a completely different setting, called external validation [64, 80, 86]. This is especially complex with high-dimensional models. It requires substantial data harmonization and technical efforts to be able to validate such models. Within the OHDSI consortium, a predictive algorithm with hundreds of predictors could be validated after harmonization of data in a common format [67]. However, this study remains one of few examples.

## Bias

As with all clinical studies, studies with big data can be biased. An important bias in these studies is selection bias, for example, arising because of inappropriate handling of missing data [87]. The majority of ML studies fail to report how missing data were handled [79, 88] or use methods (e.g., complete case) that are not recommended [62, 89, 90]. Another source of selection bias is the inappropriate exclusion of groups of patients, also a common issue in prediction studies that use ML techniques [79]. A completely different type of bias, misclassification bias, also occurs regularly in studies with big data. Examples include multilevel data with different data definitions per cluster [91, 92] or insurance claim data with only limited granularity in defining exposures and outcomes [93]. To reduce the effect of these biases, epidemiologists and statisticians have developed frameworks that are readily available in most statistical software [89, 94–96]. Probably because ML was developed in a more deterministic environment, implementations of epidemiological frameworks to ML techniques are still lacking. For clinical researchers, it might still be reasonable to use more traditional methods because right now there is more experience to address biases with these techniques (e.g., resulting from missing data).

Because bias can only be identified when a study is adequately reported, we recommend the use of reporting guidelines for prediction models (TRIPOD [97]) and the upcoming guidelines for ML techniques (TRIPOD-AI [98]). There are currently no guidelines available for clustering studies.

## Discussion

In this article, we have discussed opportunities and pitfalls of the use of ML techniques in controversial areas of (neuro)critical care research. Clustering studies are increasingly popular in critical care research. They can be used to explore new ways of describing or characterizing patient groups and suggest how patients can be treated better. A large challenge toward responsible use of these techniques is ensuring generalizability of these findings. For predictive questions, there is much discussion as to what algorithm should be used to most accurately predict outcome. The usefulness of ML techniques compared with statistical techniques depends on the volume of the data, the dimensionality of the preferred model, and the extent to which missing data or potential bias is present in the data. There are areas in which modern flexible techniques may be preferred, but efforts should be made to provide more comprehensive frameworks for using modern ML techniques.

More generally, we advocate testing research hypotheses just like clinical hypotheses are being tested. The recent uprising of large and complex data sets together with modern “data-mining” techniques has led to more data-driven hypothesis testing. Although this approach enables researchers to serendipitously encounter potentially new truths, it does overestimate the value of new data over current knowledge. It is important to be aware of prior probabilities of hypotheses being true and regard new evidence in that context [15]. Similarly, it is advised that only in patients with TBI with some risk of an intracranial lesion should the hypothesis of having such a lesion be tested using a computed tomography scan [99, 100]. An example of overestimation of the value of new data was a recent analysis in which TBI clusters were formed on the basis of two large data sets [48, 49]. Even though previous studies concluded that the GCS can “stand the test of time” [101] (i.e., remains a robust predictor of outcome), the authors conclude that their “data-derived patient phenotypes will enhance TBI patient stratification…beyond the GCS-based gold standard” [49]. They therefore provide an alternative to the GCS, thereby disregarding the long-standing use of this important characteristic. Even though a data set is large, the collective “data set” of medical knowledge being built up over centuries of meticulous research endeavors is much larger. Big data is not to be regarded as an endless source of full information about patients but rather as an opportunity to generate new hypotheses and update our knowledge.

We have not yet touched on the last-mentioned type of research question, causal questions. Causality is particularly hard to infer because it requires the application of a causal model of the problem to the data [16, 102]. A commonly used method is to include confounding factors in a regression model so that the estimated effect of the treatment on outcome is corrected for those confounding factors. This is more complex with ML techniques. Part of the enthusiasm for ML techniques stems from the idea that they require less assumptions. In fact, most ML techniques do not allow researchers to assume relationships between variables. This is, however, a drawback when exactly that control over the model is required to infer causality. Besides, the individual effects of ML techniques are relatively hard to interpret. Although more explainable algorithms exist, effects are less intuitive to interpret than regression coefficients [103]. There are some extensions of ML techniques that allow the researcher to assume relationships between variables, for example, through graph-based neural networks [104] or Bayesian networks [102, 105]. This type of control is required to appropriately address causal questions.

Some ML techniques, such as neural networks and random forests, are relatively hard to explain compared with regression models or decision trees, for example. It can be argued that algorithms should be explainable to be used in clinical practice and that clinicians and patients should be able to interpret what happens under the hood of an algorithm to ensure safety and establish control [106, 107]. However, the extent to which we can judge the reliability of an algorithm on the basis of how the algorithm arrives at its prediction is limited [108]. If the sole purpose is prediction, the relatively limited interpretability of some ML techniques may not be a problem. More relevant criteria to judge an algorithm in clinical practice might be (1) whether the algorithm shows good performance (discrimination and calibration) when validated in a relevant population [77, 80] and (2) whether relevant patient outcomes improve when the algorithm is used [63].

We suggest that there is a necessity for improved interaction between the engineer mindset of experts in ML techniques, the focus on limiting bias by epidemiologists, and the probabilistic mindset of statisticians. The difference in mindset becomes apparent when reading each professional’s published literature. For example, what is known in statistical literature as fitting or estimating is called learning or training in the ML literature [109]. Similarly, external validity is called transportability, and covariates are called features [110]. By developing a common language between these groups of researchers, we avoid complexities when aggregating results in systematic reviews and increase cross-contamination of ideas [109]. By converging these worlds, we probably will be able to extract more information from data, while avoiding harm by neglecting the need to address potential biases.

**Table:** 

Box 1. Take aways for the clinical neurocritical care researcher
• Include researchers from various backgrounds (clinical, statistical, epidemiological, data scientists) in new research projects and be more critical toward studies that only include researchers from one perspective • When reporting a prediction study, use the TRIPOD [97] or TRIPOD-AI (for ML studies, upcoming [98]) reporting guidelines so that readers can adequately assess reliability • Use only predictive algorithms in clinical practice that have been rigorously validated and that have been shown to add clinical benefit to patients when used • Appreciate the exploratory nature of clustering studies: use their results only as tentative updates on current knowledge about different patient groups rather than “new truths” (and refrain from using them in a prognostic framework)

## Conclusion

There are important pitfalls and opportunities to consider when performing clustering or predictive studies with ML techniques. More generally, we advocate to be careful as not to overvalue new data compared with clinical relevance and collective knowledge. Also, there is a necessity for improved interaction between the engineer mindset of experts in ML techniques, the focus on limiting bias by epidemiologists, and the probabilistic mindset of statisticians: we need to extract as much information from data as possible, while avoiding harm when invalid algorithms are used to inform medical decision-making.

## Supplementary Information

Below is the link to the electronic supplementary material.

Supplementary file1 (DOCX 711 kb)

## References

[1] Carteron L, Bouzat P, Oddo M (2017). Cerebral microdialysis monitoring to improve individualized neurointensive care therapy: an update of recent clinical data. Front Neurol.

[2] Hutchinson PJ, Jalloh I, Helmy A, Carpenter KLH, Rostami E, Bellander BM (2015). Consensus statement from the 2014 international microdialysis forum. Intensive Care Med.

[3] Fleuren LM, de Bruin DP, Tonutti M, Lalisang RCA, Elbers PWG, Gommers D, et al. Large-scale ICU data sharing for global collaboration: the first 1633 critically ill COVID-19 patients in the Dutch Data Warehouse. Intensive Care Med [Internet]. Springer Science and Business Media Deutschland GmbH; 2021 [cited 2021 Nov 11];47:478–81. Available from: https://link.springer.com/article/10.1007/s00134-021-06361-x 10.1007/s00134-021-06361-x PMC7887418 33595710

[4] Hyland SL, Faltys M, Hüser M, Lyu X, Gumbsch T, Esteban C, et al. Early prediction of circulatory failure in the intensive care unit using machine learning. Nat Med 2020 263 [Internet]. Nature Publishing Group; 2020 [cited 2021 Nov 11];26:364–73. Available from: https://www.nature.com/articles/s41591-020-0789-4 10.1038/s41591-020-0789-4 32152583

[5] Thoral PJ, Peppink JM, Driessen RH, Sijbrands EJG, Kompanje EJO, Kaplan L, et al. Sharing ICU Patient Data Responsibly under the Society of Critical Care Medicine/European Society of Intensive Care Medicine Joint Data Science Collaboration: The Amsterdam University Medical Centers Database (AmsterdamUMCdb) Example. Crit Care Med [Internet]. Lippincott Williams and Wilkins; 2021 [cited 2021 Nov 11];E563–77. Available from: https://journals.lww.com/ccmjournal/Fulltext/2021/06000/Sharing_ICU_Patient_Data_Responsibly_Under_the.16.aspx

[6] Fleuren LM, Dam TA, Tonutti M, de Bruin DP, Lalisang RCA, Gommers D, et al. The Dutch Data Warehouse, a multicenter and full-admission electronic health records database for critically ill COVID-19 patients. Crit Care [Internet]. BioMed Central Ltd; 2021 [cited 2021 Nov 11];25:1–12. Available from: https://link.springer.com/articles/10.1186/s13054-021-03733-z 10.1186/s13054-021-03733-z PMC8381710 34425864

[7] Saeed M, Lieu C, Raber G, Mark RG (2002). MIMIC II: a massive temporal ICU patient database to support research in intelligent patient monitoring. Comput Cardiol.

[8] Johnson AEW, Pollard TJ, Shen L, Lehman LWH, Feng M, Ghassemi M, et al. MIMIC-III, a freely accessible critical care database. Sci Data 2016 31 [Internet]. Nature Publishing Group; 2016 [cited 2021 Nov 11];3:1–9. Available from: https://www.nature.com/articles/sdata201635 10.1038/sdata.2016.35 PMC4878278 27219127

[9] Steyerberg EW, Wiegers E, Sewalt C, Buki A, Citerio G, De Keyser V, et al. Case-mix, care pathways, and outcomes in patients with traumatic brain injury in CENTER-TBI: a European prospective, multicentre, longitudinal, cohort study. Lancet Neurol. 2019;18. 10.1016/S1474-4422(19)30232-7 31526754

[10] Maas AIR, Menon DK, Steyerberg EW, Citerio G, Lecky F, Manley GT (2015). Collaborative European neurotrauma effectiveness research in traumatic brain injury (CENTER-TBI): a prospective longitudinal observational study. Neurosurgery.

[11] Nelson LD, Temkin NR, Dikmen S, Barber J, Giacino JT, Yuh E, et al. Recovery After Mild Traumatic Brain Injury in Patients Presenting to US Level I Trauma Centers A Transforming Research and Clinical Knowledge in Traumatic Brain Injury (TRACK-TBI) Study Supplemental content. JAMA Neurol [Internet]. 2019;76:1049–59. Available from: https://jamanetwork.com/10.1001/jamaneurol.2019.1313 PMC6547159 31157856

[12] Maas AIR, Menon DK, Adelson PD, Andelic N, Bell MJ, Belli A, et al. Traumatic brain injury: integrated approaches to improve prevention, clinical care, and research. Lancet Neurol [Internet]. 2017;4422. Available from: http://linkinghub.elsevier.com/retrieve/pii/S147444221730371X 10.1016/S1474-4422(17)30371-X 29122524

[13] Ramon J, Fierens D, Güiza F, Meyfroidt G, Blockeel H, Bruynooghe M (2007). Mining data from intensive care patients. Adv Eng Inf Elsevier.

[14] Friedman JH. The role of statistics in the data revolution? Int Stat Rev. Wiley Online Library; 2001;69:5–10

[15] Ioannidis JPA. Why most published research findings are false. PLoS Med [Internet]. Public Library of Science; 2005 [cited 2019 May 14];2:e124. Available from: https://dx.plos.org/10.1371/journal.pmed.0020124 10.1371/journal.pmed.0020124 PMC1182327 16060722

[16] Shmueli G (2010). To explain or to predict?. Stat Sci Inst Math Stat.

[17] Chilamkurthy S, Ghosh R, Tanamala S, Biviji M, Campeau NG, Venugopal VK (2018). Deep learning algorithms for detection of critical findings in head CT scans: a retrospective study. Lancet Elsevier.

[18] Titano JJ, Badgeley M, Schefflein J, Pain M, Su A, Cai M, et al. Automated deep-neural-network surveillance of cranial images for acute neurologic events. Nat Med [Internet]. Springer US; 2018;24:1337–41. Available from: http://dx.doi.org/10.1038/s41591-018-0147-y 10.1038/s41591-018-0147-y 30104767

[19] Lee H, Yune S, Mansouri M, Kim M, Tajmir SH, Guerrier CE, et al. An explainable deep-learning algorithm for the detection of acute intracranial haemorrhage from small datasets. Nat Biomed Eng [Internet]. Springer US; 2019;3:173–82. Available from: http://dx.doi.org/10.1038/s41551-018-0324-9 10.1038/s41551-018-0324-9 30948806

[20] Kamnitsas K, Ledig C, Newcombe VFJ, Simpson JP, Kane AD, Menon DK, et al. Efficient multi-scale 3D CNN with fully connected CRF for accurate brain lesion segmentation. Med Image Anal [Internet]. Elsevier B.V.; 2017;36:61–78. Available from: http://dx.doi.org/10.1016/j.media.2016.10.004 10.1016/j.media.2016.10.004 27865153

[21] Wong A, Otles E, Donnelly JP, Krumm A, McCullough J, DeTroyer-Cooley O, et al. External validation of a widely implemented proprietary sepsis prediction model in hospitalized patients. JAMA Intern Med [Internet]. American Medical Association; 2021 [cited 2022 Feb 19];181:1065–70. Available from: https://jamanetwork.com/journals/jamainternalmedicine/fullarticle/2781307 10.1001/jamainternmed.2021.2626 PMC8218233 34152373

[22] Sauerbrei W, Abrahamowicz M, Altman DG, le Cessie S, Carpenter J, Abrahamowicz M (2014). STRengthening analytical thinking for observational studies: the STRATOS initiative. Stat Med.

[23] Harrell FE. Regression modeling strategies [Internet]. New York, NY: Springer New York; 2001 [cited 2019 Jan 7]. Available from: http://link.springer.com/10.1007/978-1-4757-3462-1

[24] Hilbe JM. Logistic regression.

[25] Breiman L. Bagging Predictors. 1996;24:123–40.

[26] Breiman L. Random Forests [Internet]. 2001. Available from: https://link.springer.com/content/pdf/10.1023/A:1010933404324.pdf

[27] Natekin A, Knoll A. Gradient boosting machines, a tutorial. Front Neurorobot [Internet]. Frontiers; 2013 [cited 2018 Dec 21];7:21. Available from: http://journal.frontiersin.org/article/10.3389/fnbot.2013.00021/abstract 10.3389/fnbot.2013.00021 PMC3885826 24409142

[28] Burges CJC. A tutorial on support vector machines for pattern recognition [Internet]. Data Min. Knowl. Discov. 1998. Available from: https://link.springer.com/content/pdf/10.1023%2FA%3A1009715923555.pdf

[29] Polley E, Laan M van der. Super Learner In Prediction. UC Berkeley Div Biostat Work Pap Ser [Internet]. 2010 [cited 2021 Nov 29]; Available from: https://biostats.bepress.com/ucbbiostat/paper266

[30] Van Der Laan MJ, Polley EC, Hubbard AE. Super learner. Stat Appl Genet Mol Biol [Internet]. Berkeley Electronic Press; 2007 [cited 2021 Nov 29];6. Available from: https://www.degruyter.com/document/doi/10.2202/1544-6115.1309/html 10.2202/1544-6115.1309 17910531

[31] Kassambara A. Multivariate Analysis I Practical Guide To Cluster Analysis in R Unsupervised Machine Learning. [cited 2021 Nov 29]; Available from: http://www.sthda.com

[32] Goodman LA. Exploratory latent structure analysis using both identifiable and unidentifiable models. Biometrika. Oxford University Press; 1974;61:215–31.

[33] Cramér H (1945). Mathematical methods of statistics (Uppsala: Almqvist & Wiksells).

[34] Gilks WR, Thomas A, Spiegelhalter DJ (1994). A language and program for complex bayesian modelling. Stat JSTOR.

[35] Harrell FE. My journey from frequentist to Bayesian statistics | Statistical thinking [Internet]. [cited 2021 Nov 29]. Available from: https://www.fharrell.com/post/journey/

[36] Pavlou M, Ambler G, Seaman SR, Guttmann O, Elliott P, King M (2015). How to develop a more accurate risk prediction model when there are few events. BMJ.

[37] Van Calster B, van Smeden M, De Cock B, Steyerberg EW (2020). Regression shrinkage methods for clinical prediction models do not guarantee improved performance: simulation study. Stat Methods Med Res.

[38] Christodoulou E, Ma J, Collins GS, Steyerberg EW, Verbakel JY, Van Calster B. A systematic review shows no performance benefit of machine learning over logistic regression for clinical prediction models. J Clin Epidemiol [Internet]. Pergamon; 2019 [cited 2019 Mar 22];110:12–22. Available from: https://www.sciencedirect.com/science/article/pii/S0895435618310813?via%3Dihub 10.1016/j.jclinepi.2019.02.004 30763612

[39] Jain AK, Jianchang Mao, Mohiuddin KM. Artificial neural networks: a tutorial. Computer (Long Beach Calif) [Internet]. 1996 [cited 2018 Dec 20];29:31–44. Available from: http://ieeexplore.ieee.org/document/485891/

[40] Chollet F. Deep learning with Python. Simon and Schuster. 2021.

[41] Steyerberg EW. Assumptions in regression models: additivity and linearity. Clin Predict Model. Springer; 2019. p. 227–45.

[42] Nevin DG, Brohi K. Permissive hypotension for active haemorrhage in trauma. Anaesthesia. Blackwell Publishing Ltd; 2017;72:1443–8 10.1111/anae.14034 28940420

[43] Borrell LN, Samuel L (2014). Body mass index categories and mortality risk in US adults: the effect of overweight and obesity on advancing death. Am J Public Health.

[44] Czeiter E, Amrein K, Gravesteijn BY, Lecky F, Menon DK, Mondello S, et al. Blood biomarkers on admission in acute traumatic brain injury: Relations to severity, CT findings and care path in the CENTER-TBI study. EBioMedicine [Internet]. EBioMedicine; 2020 [cited 2021 Nov 25];56. Available from: https://pubmed.ncbi.nlm.nih.gov/32464528/10.1016/j.ebiom.2020.102785 PMC7251365 32464528

[45] Kent DM, Nelson J, Dahabreh IJ, Rothwell PM, Altman DG, Hayward RA. Risk and treatment effect heterogeneity: re-analysis of individual participant data from 32 large clinical trials. Int J Epidemiol [Internet]. Oxford University Press; 2016 [cited 2018 Jun 5];45:2075–88. Available from: http://www.ncbi.nlm.nih.gov/pubmed/27375287 10.1093/ije/dyw118 PMC5841614 27375287

[46] Sun X, Ioannidis JPA, Agoritsas T, Alba AC, Guyatt G. How to use a subgroup analysis users’ guides to the medical literature. JAMA—J Am Med Assoc American Medical Association; 2014. p. 405–11. 10.1001/jama.2013.285063 24449319

[47] Roberts I, Shakur-Still H, Aeron-Thomas A, Belli A, Brenner A, Chaudary MA, et al. Effects of tranexamic acid on death, disability, vascular occlusive events and other morbidities in patients with acute traumatic brain injury (CRASH-3): A randomised, placebo-controlled trial. Lancet [Internet]. The Author(s). Published by Elsevier Ltd. This is an Open Access article under the CC BY 4.0 license; 2019;394:1713–23. Available from: http://dx.doi.org/10.1016/S0140-6736(19)32233-0 10.1016/S0140-6736(19)32233-0 PMC6853170 31623894

[48] Masino AJ, Folweiler KA. Unsupervised learning with GLRM feature selection reveals novel traumatic brain injury phenotypes. 2018 [cited 2021 Nov 25]; Available from: https://arxiv.org/abs/1812.00030v1

[49] Folweiler KA, Sandsmark DK, Diaz-Arrastia R, Cohen AS, Masino AJ. Unsupervised machine learning reveals novel traumatic brain injury patient phenotypes with distinct acute injury profiles and long-term outcomes. J Neurotrauma [Internet]. Mary Ann Liebert, Inc.; 2020 [cited 2021 Nov 25];37:1431. Available from: /pmc/articles/PMC7249479/10.1089/neu.2019.6705 PMC7249479 32008422

[50] Legrand M, Phillips R V, Malenica I, Eyler L, Fong N, Martinino A, et al. Differences in clinical deterioration among three sub-phenotypes of COVID-19 patients at the time of first positive test: results from a clustering analysis. Intensive Care Med [Internet]. 2020 [cited 2021 Nov 11];47. Available from: 10.1007/s00134-020-06236-7 10.1007/s00134-020-06236-7 PMC7569095 33074342

[51] Liu Y, Li Z, Xiong H, Gao X, Wu J. Understanding of internal clustering validation measures. Proc—IEEE Int Conf Data Mining, ICDM. 2010;911–6.

[52] Halkidi M (2001). On clustering validation techniques. J Intell Inf Syst.

[53] Juhász S, Eirinaki M, Legány C, Babos A. Cluster validity measurement techniques Cite this paper Related papers A Review of Clust ering and Clust ering Qualit y Measurement IJCERT Journal Performance of k-means based Sat ellit e Image Clust ering in RGB and HSV Color Space Rajesh R Archiving t h.

[54] Halkidi M, Batistakis Y, Vazirgiannis M. Cluster validity methods. ACM SIGMOD Rec [Internet]. ACM PUB27 New York, NY, USA; 2002 [cited 2021 Nov 14];31:40–5. Available from: https://dl.acm.org/doi/abs/10.1145/565117.565124

[55] Schinkel M, Appelman B, Butler J, Schuurman A, Joost Wiersinga W. Association of clinical sub-phenotypes and clinical deterioration in COVID-19: further cluster analyses. Intensive Care Med [Internet]. 2021 [cited 2021 Nov 11]; Available from: 10.1007/s00134-021-06363-9 10.1007/s00134-021-06363-9 PMC7891487 33604760

[56] Gravesteijn B, Sewalt C, Ercole A, Akerlund C, Nelson D, Maas A, et al. Towards a new multidimensional classification of traumatic brain injury: a CENTER-TBI study. J Neurotrauma [Internet]. 2019;neu.2019.6764. Available from: https://www.liebertpub.com/doi/10.1089/neu.2019.6764

[57] Si B, Dumkrieger G, Wu T, Zafonte R, Valadka AB, Okonkwo DO, et al. Sub-classifying patients with mild traumatic brain injury: a clustering approach based on baseline clinical characteristics and 90-day and 180-day outcomes. PLoS One [Internet]. Public Library of Science; 2018 [cited 2021 Nov 11];13. Available from: /pmc/articles/PMC6040703/10.1371/journal.pone.0198741 PMC6040703 29995912

[58] Rajagopalan S, Baker W, Mahanna-Gabrielli E, Kofke AW, Balu R. Hierarchical Cluster Analysis Identifies Distinct Physiological States After Acute Brain Injury. Neurocrit Care [Internet]. 2028 [cited 2022 Feb 19]; Available from: 10.1007/s12028-021-01362-6 10.1007/s12028-021-01362-6 PMC11346511 34661861

[59] Royston P, Altman DG, Sauerbrei W. Dichotomizing continuous predictors in multiple regression: a bad idea. Stat Med Stat Med [Internet]. 2006 [cited 2018 Oct 22];25:127–41. Available from: www.interscience.wiley.com 10.1002/sim.2331 16217841

[60] Naggara O, Raymond J, Guilbert F, Roy D, Weill A, Altman DG. Analysis by categorizing or dichotomizing continuous variables is inadvisable: an example from the natural history of unruptured aneurysms. AJNR Am J Neuroradiol [Internet]. Am J Neuroradiol; 2011 [cited 2018 Oct 22];32:437–40. Available from: http://www.ncbi.nlm.nih.gov/pubmed/21330400 10.3174/ajnr.A2425 PMC8013096 21330400

[61] Steyerberg EW, Vergouwe Y (2014). Towards better clinical prediction models: seven steps for development and an ABCD for validation. Eur Heart J.

[62] Steyerberg EW. Clinical prediction models [Internet]. Cham: Springer International Publishing; 2019 [cited 2019 Nov 1]. Available from: http://link.springer.com/10.1007/978-3-030-16399-0

[63] Steyerberg E, Moons KGM, van der Windt D, Hayden J, Perel P, Schroter S (2013). Prognosis research strategy (PROGRESS) series 3: prognostic model research. PLoS Med.

[64] Moons KGM, Kengne AP, Grobbee DE, Royston P, Vergouwe Y, Altman DG, et al. Risk prediction models: II. External validation, model updating, and impact assessment. Heart [Internet]. BMJ Publishing Group Ltd and British Cardiovascular Society; 2012 [cited 2019 Aug 19];98:691–8. Available from: http://www.ncbi.nlm.nih.gov/pubmed/22397946 10.1136/heartjnl-2011-301247 22397946

[65] Harrell FE, Lee KL, Mark DB (2005). Prognostic/clinical prediction models: multivariable prognostic models: issues in developing models, evaluating assumptions and adequacy, and measuring and reducing errors. Tutor Biostat Stat Methods Clin Stud.

[66] Stiell IG, Greenberg GH, McKnight RD, Nair RC, McDowell I, Worthington JR (1992). A study to develop clinical decision rules for the use of radiography in acute ankle injuries. Ann Emerg Med.

[67] Wang Q, Reps JM, Kostka KF, Ryan PB, Zou Y, Voss EA, et al. Development and validation of a prognostic model predicting symptomatic hemorrhagic transformation in acute ischemic stroke at scale in the OHDSI network. PLoS One [Internet]. 2020;15:1–12. Available from: http://dx.doi.org/10.1371/journal.pone.0226718 10.1371/journal.pone.0226718 PMC6946584 31910437

[68] Peduzzi P, Concato J, Kemper E, Holford TR, Feinstem AR. A simulation study of the number of events per variable in logistic regression analysis. J Clin Epidemiol. 1996. 10.1016/s0895-4356(96)00236-3 8970487

[69] Van Calster B, Nieboer D, Vergouwe Y, De Cock B, Pencina MJ, Steyerberg EW. A calibration hierarchy for risk models was defined: from utopia to empirical data. J Clin Epidemiol [Internet]. Pergamon; 2016 [cited 2019 Mar 22];74:167–76. Available from: https://www.sciencedirect.com/science/article/pii/S0895435615005818?via%3Dihub 10.1016/j.jclinepi.2015.12.005 26772608

[70] Collins GS, De Groot JA, Dutton S, Omar O, Shanyinde M, Tajar A, et al. External validation of multivariable prediction models: a systematic review of methodological conduct and reporting. BMC Med Res Methodol. BioMed Central Ltd.; 2014;14. 10.1186/1471-2288-14-40 PMC3999945 24645774

[71] Shillan D, Sterne JAC, Champneys A, Gibbison B (2019). Use of machine learning to analyse routinely collected intensive care unit data: a systematic review. Crit Care Critical Care.

[72] van Smeden M, Moons KGM, de Groot JAH, Collins GS, Altman DG, Eijkemans MJC, et al. Sample size for binary logistic prediction models: Beyond events per variable criteria. Stat Methods Med Res [Internet]. SAGE Publications Ltd; 2019 [cited 2021 Nov 14];28:2455–74. Available from: https://journals.sagepub.com/doi/full/10.1177/0962280218784726 10.1177/0962280218784726 PMC6710621 29966490

[73] Riley RD, Debray TPA, Collins GS, Archer L, Ensor J, van Smeden M, et al. Minimum sample size for external validation of a clinical prediction model with a binary outcome. Stat Med [Internet]. John Wiley & Sons, Ltd; 2021 [cited 2021 Nov 14];40:4230–51. Available from: https://onlinelibrary.wiley.com/doi/full/10.1002/sim.9025 10.1002/sim.9025 34031906

[74] van der Ploeg T, Austin PC, Steyerberg EW. Modern modelling techniques are data hungry: a simulation study for predicting dichotomous endpoints. BMC Med Res Methodol [Internet]. BioMed Central; 2014 [cited 2018 Jun 5];14:137. Available from: http://www.ncbi.nlm.nih.gov/pubmed/25532820 10.1186/1471-2288-14-137 PMC4289553 25532820

[75] Zhang W, Tang Y, Liu H, Yuan LP, Wang CC, Chen SF, et al. Risk prediction models for intensive care unitacquired weakness in intensive care unit patients: a systematic review. PLoS One [Internet]. 2021;16:1–14. Available from: http://dx.doi.org/10.1371/journal.pone.0257768 10.1371/journal.pone.0257768 PMC8462700 34559850

[76] Steyerberg EW, Eijkemans MJC, Harrell FE, Habbema JDF. Prognostic modeling with logistic regression analysis: In search of a sensible strategy in small data sets. Med Decis Mak [Internet]. SAGE Publications Inc.; 2001 [cited 2021 Nov 14];21:45–56. Available from: https://journals.sagepub.com/doi/abs/10.1177/0272989X0102100106?casa_token=Lrav0bOscOsAAAAA%3ACMoEqA2RwEW2_PWRoctJniTDiI3D9eNYyvXOyQlxyAjhXKx0NRZlWUt6uAet7CWhUlfY7XQ_7fVDSg 10.1177/0272989X0102100106 11206946

[77] Gravesteijn BY, Nieboer D, Ercole A, Lingsma HF, Nelson D, van Calster B, et al. Machine learning algorithms performed no better than regression models for prognostication in traumatic brain injury. J Clin Epidemiol [Internet]. 2020;122:95–107. Available from: https://linkinghub.elsevier.com/retrieve/pii/S0895435619308753 10.1016/j.jclinepi.2020.03.005 32201256

[78] van Os HJA, Ramos LA, Hilbert A, van Leeuwen M, van Walderveen MAA, Kruyt ND, et al. Predicting outcome of endovascular treatment for acute Ischemic stroke: potential value of machine learning algorithms. Front Neurol [Internet]. Frontiers; 2018 [cited 2019 Jan 7];9:784. Available from: https://www.frontiersin.org/article/10.3389/fneur.2018.00784/full 10.3389/fneur.2018.00784 PMC6167479 30319525

[79] Navarro CLA, Damen JAA, Takada T, Nijman SWJ, Dhiman P, Ma J, et al. Risk of bias in studies on prediction models developed using supervised machine learning techniques: systematic review. 10.1136/bmj.n2281 PMC8527348 34670780

[80] Steyerberg EW, Harrell FE. Prediction models need appropriate internal, internal-external, and external validation. J Clin Epidemiol [Internet]. 2016 [cited 2019 Mar 23];69:245–7. Available from: https://www-ncbi-nlm-nih-gov.eur.idm.oclc.org/pmc/articles/PMC5578404/pdf/nihms895839.pdf 10.1016/j.jclinepi.2015.04.005 PMC5578404 25981519

[81] Plate JDJ, Van De Leur RR, Leenen LPH, Hietbrink F, Peelen LM, Eijkemans MJC. Incorporating repeated measurements into prediction models in the critical care setting: a framework, systematic review and meta-analysis. BMC Med Res Methodol. BMC Medical Research Methodology; 2019;19:1–11. 10.1186/s12874-019-0847-0 PMC6815391 31655567

[82] Schober P, Vetter TR (2018). Repeated measures designs and analysis of longitudinal data: If at first you do not succeed-try, try again. Anesth Analg.

[83] Baart SJ, van der Palen RLF, Putter H, Tsonaka R, Blom NA, Rizopoulos D, et al. Joint modeling of longitudinal markers and time-to-event outcomes: an application and tutorial in patients after surgical repair of transposition of the great arteries. Circ Cardiovasc Qual Outcomes, 2021. 10.1161/CIRCOUTCOMES.120.007593 PMC8598112 34674542

[84] Venema E, Wessler BS, Paulus JK, Salah R, Raman G, Leung LY, et al. Large-scale validation of the prediction model risk of bias assessment Tool (PROBAST) using a short form: high risk of bias models show poorer discrimination. J Clin Epidemiol [Internet]. Elsevier Inc.; 2021;138:32–9. Available from: 10.1016/j.jclinepi.2021.06.017 10.1016/j.jclinepi.2021.06.017 34175377

[85] Steyerberg EW, Harrell FE, Borsboom GJJM, Eijkemans MJ, Vergouwe Y, Habbema JDF. Internal validation of predictive models: efficiency of some procedures for logistic regression analysis. J Clin Epidemiol [Internet]. 2001 [cited 2019 Nov 12];54:774–81. Available from: https://linkinghub.elsevier.com/retrieve/pii/S0895435601003419 10.1016/s0895-4356(01)00341-9 11470385

[86] Steyerberg EW. Clinical prediction models [Internet]. New York, NY: Springer New York; 2009 [cited 2019 Jan 7]. Available from: http://link.springer.com/10.1007/978-0-387-77244-8

[87] Wolff RF, Moons KGM, Riley RD, Whiting PF, Westwood M, Collins GS (2019). PROBAST: a tool to assess the risk of bias and applicability of prediction model studies. Ann Intern Med.

[88] Nijman S, Leeuwenberg A, Beekers I, Verkouter I, Jacobs J, Bots M, et al. Missing data is poorly handled and reported in prediction model studies using machine learning: a literature review. J Clin Epidemiol [Internet]. Pergamon; 2021 [cited 2021 Dec 5]; Available from: https://linkinghub.elsevier.com/retrieve/pii/S0895435621003759 10.1016/j.jclinepi.2021.11.023 34798287

[89] Gravesteijn BY, Sewalt CA, Venema E, Nieboer D, Steyerberg EW. Missing data in prediction research: a five-step approach for multiple imputation, illustrated in the CENTER-TBI Study. https://home.liebertpub.com/neu [Internet]. Mary Ann Liebert, Inc., publishers 140 Huguenot Street, 3rd Floor New Rochelle, NY 10801 USA ; 2021 [cited 2021 Nov 14];38:1842–57. Available from: https://www.liebertpub.com/doi/abs/10.1089/neu.2020.7218 10.1089/neu.2020.7218 33470157

[90] Molenberghs G, Kenward M, Ebrahim GJ. Missing Data in Clinical Studies. J Trop Pediatr [Internet]. Oxford University Press; 2007 [cited 2018 Oct 17];53:294–294. Available from: https://academic.oup.com/tropej/article-lookup/doi/10.1093/tropej/fmm053

[91] Young JC, Conover MM, Jonsson FM (2018). Measurement error and misclassification in electronic medical records: methods to mitigate bias. Curr Epidemiol Reports Current Epidemiol Reports.

[92] Valkhoff VE, Coloma PM, Masclee GMC, Gini R, Innocenti F, Lapi F (2014). Validation study in four health-care databases: upper gastrointestinal bleeding misclassification affects precision but not magnitude of drug-related upper gastrointestinal bleeding risk. J Clin Epidemiol.

[93] Jonsson Funk M, Landi SN (2014). Misclassification in administrative claims data: quantifying the impact on treatment effect estimates. Curr Epidemiol Rep.

[94] van Smeden M, Penning de Vries BBL, Nab L, Groenwold RHH. Approaches to addressing missing values, measurement error and confounding in epidemiologic studies. J Clin Epidemiol [Internet]. Elsevier BV; 2020 [cited 2020 Dec 1];0. Available from: 10.1016/j.jclinepi.2020.11.006 10.1016/j.jclinepi.2020.11.006 33176189

[95] Little RJA, Rubin DB. Statistical analysis with missing data. J Educ Stat [Internet]. 1991 [cited 2018 Sep 21];16:150. Available from: https://www.jstor.org/stable/1165119?origin=crossref

[96] Buuren S van. Flexible imputation of missing data. CRC Press, 2018.

[97] Leisman DE, Harhay MO, Lederer DJ, Abramson M, Adjei AA, Bakker J, et al. Development and reporting of prediction models: guidance for authors from editors of respiratory, sleep, and critical care journals. Crit Care Med [Internet]. 2020 [cited 2020 Mar 19];1. Available from: http://www.ncbi.nlm.nih.gov/pubmed/32141923 10.1097/CCM.0000000000004246 PMC7161722 32141923

[98] Collins GS, Dhiman P, Andaur Navarro CL, Ma J, Hooft L, Reitsma JB (2021). Protocol for development of a reporting guideline (TRIPOD-AI) and risk of bias tool (PROBAST-AI) for diagnostic and prognostic prediction model studies based on artificial intelligence. BMJ Open.

[99] Foks KA, Van Den Brand CL, Lingsma HF, Van Der Naalt J, Jacobs B, De Jong E, et al. External validation of computed tomography decision rules for minor head injury: prospective, multicentre cohort study in the Netherlands. BMJ. BMJ Publishing Group; 2018;362. 10.1136/bmj.k3527 PMC6108278 30143521

[100] (UK) NCGC. Head Injury. Head Inj Triage, Assessment, Investig Early Manag Head Inj Child Young People Adults [Internet]. National Institute for Health and Care Excellence (UK); 2014 [cited 2022 Feb 5]; Available from: https://www.ncbi.nlm.nih.gov/books/NBK248061/

[101] Teasdale G, Maas A, Lecky F, Manley G, Stocchetti N, Murray G (2014). The glasgow coma scale at 40 years: standing the test of time. Lancet Neurol.

[102] Pearl J, Mackenzie D. The book of why: the new science of cause and effect. Basic Books, 2018.

[103] Thoral PJ, Fornasa M, de Bruin DP, Tonutti M, Hovenkamp H, Driessen RH (2021). Explainable machine learning on AmsterdamUMCdb for ICU discharge decision support: uniting intensivists and data scientists. Crit Care Explor.

[104] Battaglia PW, Hamrick JB, Bapst V, Sanchez-Gonzalez A, Zambaldi V, Malinowski M, et al. Relational inductive biases, deep learning, and graph networks. 2018 [cited 2022 Feb 7]; Available from: https://arxiv.org/abs/1806.01261v3

[105] Heckerman D, Geiger D, Chickering DM (1995). Learning Bayesian networks : the combination of knowledge and statistical data A Bayesian network is an annotated directed graph that encodes probabilistic relationships. Mach Learn.

[106] Tonekaboni S, Joshi S, McCradden MD, Goldenberg A. What clinicians want: contextualizing explainable machine learning for clinical end use. 2019;1–21. Available from: http://arxiv.org/abs/1905.05134

[107] Cutillo CM, Sharma KR, Foschini L, Kundu S, Mackintosh M, Mandl KD, et al. Machine intelligence in healthcare—perspectives on trustworthiness, explainability, usability, and transparency. npj Digit Med. 2020;3:1–5. 10.1038/s41746-020-0254-2 PMC7099019 32258429

[108] Ghassemi M, Oakden-Rayner L, Beam AL. The false hope of current approaches to explainable artificial intelligence in health care. Lancet Digit Heal [Internet]. The Author(s). Published by Elsevier Ltd. This is an Open Access article under the CC BY 4.0 license; 2021;3:e745–50. Available from: http://dx.doi.org/10.1016/S2589-7500(21)00208-9 10.1016/S2589-7500(21)00208-9 34711379

[109] Faes L, Sim DA, van Smeden M, Held U, Bossuyt PM, Bachmann LM (2022). Artificial intelligence and statistics: just the old wine in new Wineskins?. Front Digit Heal.

[110] Machine learning versus traditional statistical modeling and medical [Internet]. [cited 2022 Feb 19]. Available from: https://de.slideshare.net/MaartenvanSmeden/machine-learning-versus-traditional-statistical-modeling-and-medical-doctors

[111] Austin PC, Harrell FE, Steyerberg EW (2021). Predictive performance of machine and statistical learning methods: impact of data-generating processes on external validity in the “large N, small p” setting. Stat Methods Med Res.

[112] Harrell FE, Habbema JDF, Steyerberg EW, Eijkemans MJC (2000). Prognostic modelling with logistic regression analysis: a comparison of selection and estimation methods in small data sets. Stat Med.

