# Supplementary material for: Modern Learning from Big Data in Critical Care: Primum Non Nocere
Source: Neurocrit Care. 2022 May 5;37(Suppl 2):174–84. doi: 10.1007/s12028-022-01510-6 (PMC9071245; doi:10.1007/s12028-022-01510-6)
Supplement: Supplementary file 1 — Supplementary file1 (DOCX 711 kb) [file 12028_2022_1510_MOESM1_ESM.docx]

# Appendix A

In this appendix, methodological terms are explained in more detail than in the main manuscript.

## Validation

Validation is the process of evaluating whether research findings generalize to a new setting. Although this is an important factor in research in general, this is particularly important in predictive and clustering studies [1,2]. It is important for any studies that uses either machine learning or more traditional statistical methods [3].

For predictive research, validation is essentially performance assessment. This is especially important because performance assessments in development studies are often too optimistic. When an algorithm is applied to a new dataset in a different setting, the performance is often lower [4]. This is important for when the model is to be used on new patients who come from different contexts than in the development study. If more flexible models are used, more parameters need to be estimated from the data. Therefore, their performance is more uncertain in new contexts [5,6]. These models need proper validation.

For clustering studies, validation is important because the distribution of characteristics in a particular study might be different than in actual clinical practice. The distinguished groups might appear more relevant than they actually are in clinical practice.

### Internal validation

We refer to the validation of an algorithm within the same setting as internal validation [5]. Clinicians should be cautious to use a model that has only been internally validated. In different settings, the model might perform worse, and can provide miscalibrated predictions.

## Split sample validation

Split-sample validation is a common approach to internal validation. The data is split at random in a *train* and *test* set. The algorithm is developed on the *train* set, and then applied on the *test* set. Within the test set, performance of the algorithm can be calculated (see “performance”, later on). Although this method is simple, it is inefficient. Both model development and the estimated performance are unstable, since result heavily rely on where the data is splitted [7–9].

### Cross-validation

A more robust way of internally validating a prediction model is cross-validation (figure 1). Iteratively, models are developed on one part of the dataset, and the performance is estimated on the part that was left out. The average of the performances is less likely to rely on where the data is splitted and is more efficient [8].


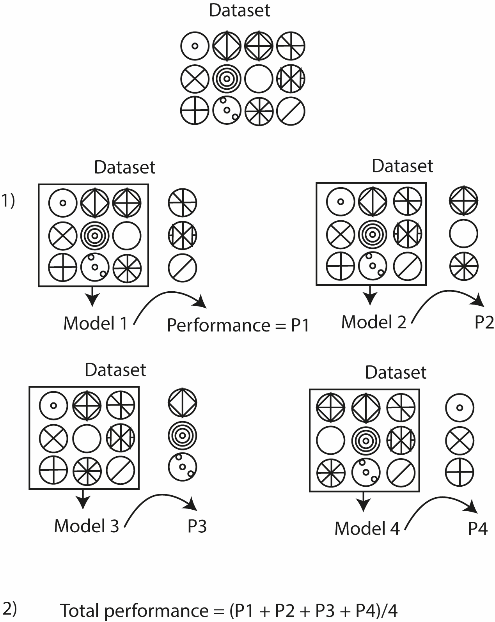


Figure 1, diagram showing an example for cross-validation. In this example, a 4-time crossvalidation is performed on a dataset with 12 individuals (step 1). The total performance is the mean of the performances measured in step 1 (step 2). The standard error of the total performance can be calculated using for example Rubin’s Rules [10,11].

### Bootstrapping

Another way of internally validating an algorithm is using a bootstrap method (figure 2). This method relies on sampling with replacement. It is even more accurate and efficient than a cross-validation method [8]. Some do find the method somewhat harder to implement.


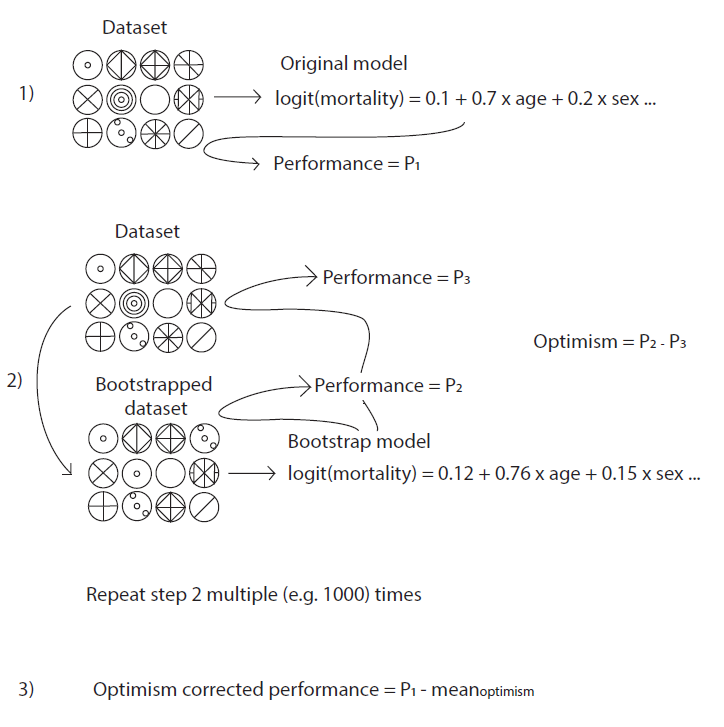


Figure 2, diagram that shows how bootstrap validation should be performed for internal validation. Adapted from Gravesteijn et al. [10]

### External validation

The most rigorous way of validation is when the algorithm is evaluated in a new dataset from a context different than the original dataset. This is referred to as external validation [5].

### Internal-external validation

Finally, it is possible to perform cross-validation with a specific rule for selecting the developmental and test set [1]. For example, it is possible to cross-validate prediction models by developing the model on all but one center in an individual patient data meta-analysis [3,12]. Heterogeneity in performance can be explored, which is a benefit of performing such internal-external validation. An example is provided in Riley et al. [13]: the QRISK2 shows lower discrimination in studies with higher percentage of smokers. This potentially implies that the model performs worse in smokers, than in non-smokers. This impacts how the model should be applied in clinical practice.

## Performance

Performance of a model is often calculated by comparing the calculated risks in new individuals with the actual outcomes that have occurred (for prognostic models). Although many metrics exist, key measures are related to discrimination and/or calibration [14,15]. We explain these two metrics below.

### Discrimination

Discrimination refers to the ability of the model to distinguish patients with and without the predicted outcome [16]. It is commonly reported with Harrell’s Concordance (C) statistic, equivalent to the area under the ROC curve, which can be interpreted as follows: it is the probability that in two randomly sampled individuals, one with and one without the outcome, the algorithm predicts a higher risk for the patient with the outcome [17]. A C-statistic close to 1 indicates almost perfect discrimination, and close to 0.5 not much better than a coin-flip.

### Calibration

Calibration refers to the extent that the model predicts an adequate risk score for patients, e.g.: in 100 patients with a predicted mortality risk of 10%, do 10 patients actually die on average? To assess calibration, we advocate the use of calibration curves (figure 1) [18]. Calibration is essential for reliable support for clinical decisions [19]: if the predicted risk is markedly different than actual risk, alternative treatment decisions might actually harm patients. For example, It is recommended to start statin treatment in patient with high cholesterol and >10% risk on cardiovascular death [20]. If predicted risk is lower than actual risk around the treatment threshold (figure 1), patients that have a risk of 10% might not receive treatment that could have prevented cardiovascular mortality because their predicted risk is too low.


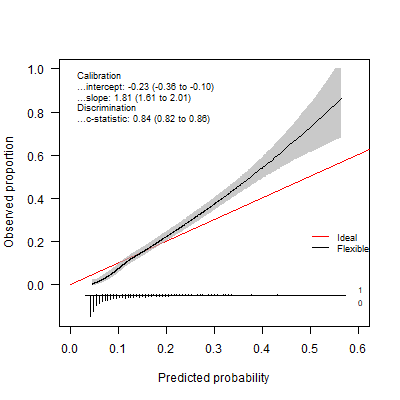


Figure 3, calibration curve fitted to simulated data. The x-axis shows the (simulated) risk on cardiovascular mortality in 3000 patients, the y-axis shows the (simulated) observed outcomes in this group. The model is miscalibrated: the predictions are too close to the event rate (10%). Therefore, around the treatment threshold (10%), the predicted risk is too low. This severe miscalibration does not affect discrimination here: the discrimination of the model is still good (C-statistic of 0.84, 95% CI: 0.82 – 0.86).

## References

1. Steyerberg EW, Harrell FE. Prediction models need appropriate internal, internal-external, and external validation. J Clin Epidemiol [Internet]. 2016 [cited 2019 Mar 23];69:245–7. Available from: https://www-ncbi-nlm-nih-gov.eur.idm.oclc.org/pmc/articles/PMC5578404/pdf/nihms895839.pdf

2. Collins GS, De Groot JA, Dutton S, Omar O, Shanyinde M, Tajar A, et al. External validation of multivariable prediction models: A systematic review of methodological conduct and reporting. BMC Med Res Methodol. BioMed Central Ltd.; 2014;14.

3. Gravesteijn BY, Nieboer D, Ercole A, Lingsma HF, Nelson D, van Calster B, et al. Machine learning algorithms performed no better than regression models for prognostication in traumatic brain injury. J Clin Epidemiol [Internet]. 2020;122:95–107. Available from: https://linkinghub.elsevier.com/retrieve/pii/S0895435619308753

4. Steyerberg EW, Vickers AJ, Cook NR, Gerds T, Gonen M, Obuchowski N, et al. Assessing the performance of prediction models: a framework for traditional and novel measures. Epidemiology [Internet]. NIH Public Access; 2010 [cited 2019 Feb 18];21:128–38. Available from: http://www.ncbi.nlm.nih.gov/pubmed/20010215

5. Steyerberg EW. Clinical Prediction Models [Internet]. Cham: Springer International Publishing; 2019 [cited 2019 Nov 1]. Available from: http://link.springer.com/10.1007/978-3-030-16399-0

6. Harrell FE. Regression Modeling Strategies [Internet]. New York, NY: Springer New York; 2001 [cited 2019 Jan 7]. Available from: http://link.springer.com/10.1007/978-1-4757-3462-1

7. Steyerberg EW, Vergouwe Y. Towards better clinical prediction models: seven steps for development and an ABCD for validation. Eur Heart J [Internet]. Oxford University Press; 2014 [cited 2018 Dec 21];35:1925–31. Available from: https://academic.oup.com/eurheartj/article-lookup/doi/10.1093/eurheartj/ehu207

8. Steyerberg EW, Harrell FE, Borsboom GJJM, Eijkemans MJ., Vergouwe Y, Habbema JDF. Internal validation of predictive models: Efficiency of some procedures for logistic regression analysis. J Clin Epidemiol [Internet]. 2001 [cited 2019 Nov 12];54:774–81. Available from: https://linkinghub.elsevier.com/retrieve/pii/S0895435601003419

9. Steyerberg EW. Validation in prediction research: the waste by data splitting. J Clin Epidemiol [Internet]. Elsevier Inc.; 2018;103:131–3. Available from: https://doi.org/10.1016/j.jclinepi.2018.07.010

10. Gravesteijn BY, Sewalt CA, Venema E, Nieboer D, Steyerberg EW. Missing Data in Prediction Research: A Five-Step Approach for Multiple Imputation, Illustrated in the CENTER-TBI Study. https://home.liebertpub.com/neu [Internet]. Mary Ann Liebert, Inc., publishers 140 Huguenot Street, 3rd Floor New Rochelle, NY 10801 USA ; 2021 [cited 2021 Nov 14];38:1842–57. Available from: https://www.liebertpub.com/doi/abs/10.1089/neu.2020.7218

11. Rubin DB, Schenker N. Multiple imputation in health-are databases: An overview and some applications. Stat Med [Internet]. 1991 [cited 2019 Nov 28];10:585–98. Available from: http://doi.wiley.com/10.1002/sim.4780100410

12. Steyerberg EW, Nieboer D, Debray TPA, Houwelingen HC. Assessment of heterogeneity in an individual participant data meta‐analysis of prediction models: An overview and illustration. Stat Med [Internet]. John Wiley and Sons Ltd; 2019 [cited 2020 Nov 27];38:4290–309. Available from: https://onlinelibrary.wiley.com/doi/abs/10.1002/sim.8296

13. Riley RD, Ensor J, Snell KIE, Debray TPA, Altman DG, Moons KGM, et al. External validation of clinical prediction models using big datasets from e-health records or IPD meta-analysis: Opportunities and challenges. BMJ. 2016;353:27–30.

14. Steyerberg EW, Pencina MJ, Lingsma HF, Kattan MW, Vickers AJ, Van Calster B. Assessing the incremental value of diagnostic and prognostic markers: a review and illustration. Eur J Clin Invest [Internet]. 2012 [cited 2019 Sep 23];42:216–28. Available from: https://onlinelibrary.wiley.com/doi/10.1111/j.1365-2362.2011.02562.x

15. Steyerberg EW, Vergouwe Y. Towards better clinical prediction models: Seven steps for development and an ABCD for validation. Eur Heart J. 2014;35:1925–31.

16. Royston P, Altman DG. Visualizing and assessing discrimination in the logistic regression model. Stat Med. 2010;29:2508–20.

17. Harrell FE, Califf RM, Pryor DB, Lee KL, Rosati RA. Evaluating the Yield of Medical Tests. JAMA J Am Med Assoc. 1982;247:2543–6.

18. Van Calster B, Nieboer D, Vergouwe Y, De Cock B, Pencina MJ, Steyerberg EW. A calibration hierarchy for risk models was defined: from utopia to empirical data. J Clin Epidemiol [Internet]. Pergamon; 2016 [cited 2019 Mar 22];74:167–76. Available from: https://www.sciencedirect.com/science/article/pii/S0895435615005818?via%3Dihub

19. Van Calster B, McLernon DJ, Van Smeden M, Wynants L, Steyerberg EW, Bossuyt P, et al. Calibration: The Achilles heel of predictive analytics. BMC Med. BMC Medicine; 2019;17:1–7.

20. Mach F, Baigent C, Catapano AL, Koskinas KC, Casula M, Badimon L, et al. 2019 ESC/EAS Guidelines for the management of dyslipidaemias: Lipid modification to reduce cardiovascular risk. Eur Heart J. 2020;41:111–88.
